# Endothelial Focal Adhesions Are Functional Obstacles for Leukocytes During Basolateral Crawling

**DOI:** 10.3389/fimmu.2021.667213

**Published:** 2021-05-18

**Authors:** Janine J. G. Arts, Eike K. Mahlandt, Lilian Schimmel, Max L. B. Grönloh, Sanne van der Niet, Bart J. A. M. Klein, Mar Fernandez-Borja, Daphne van Geemen, Stephan Huveneers, Jos van Rijssel, Joachim Goedhart, Jaap D. van Buul

**Affiliations:** ^1^ Molecular Cell Biology Lab, Department of Molecular Hematology, Sanquin Research and Landsteiner Laboratory, Amsterdam, Netherlands; ^2^ Leeuwenhoek Centre for Advanced Microscopy (LCAM), Section of Molecular Cytology, Swammerdam Institute for Life Sciences (SILS), University of Amsterdam, Amsterdam, Netherlands; ^3^ Department of Medical Biochemistry, Amsterdam Cardiovascular Sciences, Amsterdam University Medical Center (UMC), University of Amsterdam, Amsterdam, Netherlands

**Keywords:** transmigration, focal adhesions, small GTPase, RhoJ, Tiam1, endothelium, inflammation

## Abstract

An inflammatory response requires leukocytes to migrate from the circulation across the vascular lining into the tissue to clear the invading pathogen. Whereas a lot of attention is focused on how leukocytes make their way through the endothelial monolayer, it is less clear how leukocytes migrate underneath the endothelium before they enter the tissue. Upon finalization of the diapedesis step, leukocytes reside in the subendothelial space and encounter endothelial focal adhesions. Using TIRF microscopy, we show that neutrophils navigate around these focal adhesions. Neutrophils recognize focal adhesions as physical obstacles and deform to get around them. Increasing the number of focal adhesions by silencing the small GTPase RhoJ slows down basolateral crawling of neutrophils. However, apical crawling and diapedesis itself are not affected by RhoJ depletion. Increasing the number of focal adhesions drastically by expressing the Rac1 GEF Tiam1 make neutrophils to avoid migrating underneath these Tiam1-expressing endothelial cells. Together, our results show that focal adhesions mark the basolateral migration path of neutrophils.

## Introduction

Neutrophils in the bloodstream form the first line of defense during an infection. To fulfil their function at the site of inflammation, they must exit the vasculature in a process referred to as transendothelial migration (TEM). To prevent vascular leakage, endothelial cells lining the blood vessels form a tight barrier involving the adherens junction protein VE-cadherin. During inflammation, these junctions allow leukocytes to cross while keeping the barrier intact. The endothelium is activated by pro-inflammatory stimuli, resulting in the upregulation of adhesion molecules such as selectins, ICAM-1 and VCAM-1. Neutrophils slowdown from the circulation and after the multistep process of rolling, adhesion, crawling and diapedesis they enter the subendothelial space (1, 2). Once crossed the endothelium, neutrophils tend to stay between the endothelium and the basement membrane for up to 20 minutes before penetrating the inflamed tissue (3, 4). It seems that they are actively searching for a spot to enter the tissue. However, the factors that control the crawling of neutrophils at the basolateral level are not well known. Identifying these factors may provide new therapeutic options to promote or reduce tissue infiltration of immune cells.

TEM mainly occurs in post capillary venules which consist of an endothelial cell layer, supporting pericytes and a basement membrane. Endothelial cells are physically attached to the underlying basement membrane through protein complexes called focal adhesions (FAs), linking the actin cytoskeleton to the basement membrane (5). FAs assemble when integrin receptors are activated by the extracellular matrix, resulting in recruitment of multiple FA proteins as paxillin, vinculin, Focal Adhesion Kinase (FAK) and talin (6). Nascent adhesions can rapidly disassemble or mature to focal complexes. Thereafter, focal complexes mature into FAs that are larger and reside at the end of actin fibers (7). The assembly and disassembly of FAs together with the remodeling of the actin cytoskeleton enable cells to migrate and to maintain their barrier function. *En face* imaging of human vascular endothelial beds from different tissues showed the presence of these complexes in arteries as well as venules (8).

It was reported that T-cell TEM preferentially occurred through junctions where near-junction FA density of ECs was low (9). Moreover, the disruption of adhesions was observed, and a transient weakening of the FAs was hypothesized to be necessary to widen subendothelial spaces. Another study showed that disruption of FAK protein or FAK signaling decreased neutrophil transmigration (5). However, if FA dynamics are involved in the efficiency how neutrophils maneuver underneath the endothelium is not known.

The remodeling and turnover of FAs involves the small GTPases RhoJ (10, 11), which has been shown to be expressed in the vasculature *in vivo* (12, 13). RhoJ is a member of the family of Rho GTPases acting as molecular switches cycling from an inactive GDP-bound mode to an active GTP-bound mode. Guanine exchange factors (GEFs) and GTPase activating proteins (GAPs) assist in this switching. Rho GTPases mainly regulate the cell’s cytoskeleton, thereby fulfilling a major role in cellular homeostasis. Although several studies have focused on the contribution of the endothelial GTPases RhoA, Cdc42 and Rac1 on leukocyte TEM (14–20), nothing is known about the role of the FA-regulating RhoJ in leukocyte TEM.

Here, we show that neutrophils underneath the endothelium navigate around FAs as physical obstacles. Depletion of RhoJ increased the number of FAs, resulting in a reduction of the speed and motility of basolateral crawling of neutrophils. RhoJ perturbation did not alter apical rolling, crawling or diapedesis of neutrophil TEM. Furthermore, by drastically increasing the number of FAs by expressing an active mutant of the Rac1 GEF Tiam1 (C1199), we were able to steer neutrophil basolateral crawling underneath endothelial cells that displayed lowest number of FAs. This supports our hypothesis that FAs can mark the path for basolateral migration of neutrophils.

## Materials and Methods

### Cell Culture

Human umbilical vein endothelial cells (HUVECs) (Lonza) were cultured in EGM2 medium (Cat.nr C-22110, Promocell) supplemented with 1% penstrep (Invitrogen). The culture flasks, plates (TPP) and coverslips were coated with fibronectin (30 µg/ml; Sanquin Reagents, Amsterdam, Netherlands). HUVECs were used during experiments when at passage 3-4. Blood-outgrowth Endothelial Cells (BOECs) were isolated as described before (21) and cultured in EGM2 medium (Cat.nr C-22110, Promocell) supplemented with 1% penstrep (Invitrogen) and 18% fetal calf serum (Bodinco). The culture flasks, plates (TPP) and coverslips were coated with gelatin (Sigma). Endothelial cells were cultured at 37°C and 5% CO2.

### Umbilical Cord

Human umbilical cords were collected from the Department of Obstetrics and Gynaecology of the Amsterdam UMC (VUMC) after obtaining written informed consent from the mothers. 1 to 2-day old human umbilical cords were used for all our experiments. The outer ends of the cord were cut off. A cannula was inserted in the human umbilical vein and it was tied up with a tie-rip. The vein was gently flushed with warm PBS to remove blood and blood clots. Then the vein was either fixed or stained directly. Alternatively, HUVECs were removed (denudation) by 0.05% trypsin-EDTA solution (Sigma cat #59418C) and rinsed with PBS. The vein was cut in pieces of approximately 1 cm^2^ and 250.000 BOECs were seeded on the vein in a 12-well plate. After overnight incubation 37°C and 5% CO_2 _and 4 hours of TNF-alpha stimulation (10 ng/ml, Peprotech) veins with BOEC were fixed and stained.

### Immunofluorescence Staining

Umbilical cords: Umbilical veins were fixed with 4% PFA (Merck Millipore, 104005) in PBS containing 1 mM CaCl_2_ and 0.5 mM MgCl_2_ for 15 minutes. Veins were washed with PBS and quenched with 50 mM NH_4_Cl in PBS for 10 minutes at RT. Veins were then permeabilized with 0.5% Triton X-100 (Sigma, USA, 9002-93-1) for 5 minutes. Subsequently, unspecific binding sites were blocked with 1% bovine serum albumin (BSA) (PAA laboratories, Austria, Pasching, AFFY10857) in 50mM NH_4_Cl in PBS++ for 60 minutes at RT. For immunostaining, 50 µL of primary antibody solution in PBS-BSA was added to the luminal side of the vein and incubated for 60 minutes at RT. After washing with PBS, the veins were incubated with fluorescently labelled secondary antibodies for 60 minutes at RT. After washing with PBS, veins were mounted with the luminal surface facing down on glass-bottom Petri dishes using DAPI Fluoromount-G mounting medium (Southern Biotech, cat #0100-20). For the mesenteric arteries, we used the protocol as described by van Geemen et al. (8). HUVECs were cultured on fibronectin coated coverslips (12mm, Thermo Scientific). Cells were fixed with 4% paraformaldehyde in PBS containing 1mM CaCl_2_ and 0.5 mM MgCl_2_ for 10 minutes and washed three times. Thereafter, HUVECs were permeabilized with 0.1% Triton-X 100 in PBS containing 1mM CaCl_2_ and 0.5 mM MgCl_2_ for 5 minutes and blocked using 2% BSA in PBS containing 1mM CaCl_2_ and 0.5 mM MgCl_2_ for 30 minutes. Coverslips were incubated with primary antibodies for one hour and washed three times, followed by 1 hour incubation with secondary antibodies. After three times washing, the coverslips were mounted using Mowiol (10% Mowiol^®^4-88, 2.5% Dabco, 25% Glycerol, pH 8.5). For the lattice light sheet microscopy imaging and processing, we refer to (22).

### Antibodies and Imaging

Polyclonal rabbit antibody against phospho-Paxillin (cat# 44-722G), Alexa 488-conugated chicken anti rabbit (cat# A21441) secondary antibody, Alexa 633-conjugated goat anti mouse (cat# A21050) secondary antibody and Phalloidin-Texas Red (cat# T7471) were purchased from Invitrogen. Mouse monoclonal antibody against Fibronectin (cat# 610077) and Alexa 647-conjugated mouse monoclonal antibody against VE-cadherin (cat# 561567) were purchased from BD biosciences. Hoechst 33342 (cat# H-1399) was purchased from Molecular Probes. Goat Polyclonal anti VE-cadherin antibody was purchased from Santa Cruz (cat #SC-6458). Secondary Chicken anti Goat Alexa 647 conjugated antibody was purchased from Invitrogen (cat#A21469). Polyclonal rabbit antibody against Laminin 1αβγ and 2 αβγ was purchased from Novus Biologicals (cat# NB300-177). Monoclonal mouse antibody against Laminin α4 was purchased from R&D Systems (cat# MAB7340). Polyclonal rabbit antibody against Collagen IV was purchased from Abcam (cat# ab6586). Polyclonal rabbit antibody against HA was purchased from Sigma (cat# H6908). Images were acquired using a confocal laser scanning microscope (Leica SP8) using a 20x, 40x, or 63x NA 1.4 oil immersion objective and HyD detectors.

### Plasmids

mNeonGreen-Paxillin is available through Addgene (plasmid # 129604) (23). mNeonGreen-Paxillin was PCR amplified with the following primers: FW 5’-AGGTCTATATAAGCAGAGC-3’, RV 5’-ATATGCTAGCCTAGCAGAAGAGCTTGAGGAAG-3’. PCR product and lentiviral backbone were restriction digested with AgeI and NheI. The fragments were ligated to create pLV-mNeonGreen-Paxillin. Tiam1-C1199-HA constitutively active mutant was a kind gift of John Collard and was microporated into HUVEC using Neon Transfection System (ThermoFisher) according to manufacturer’s protocol (HA tag from Human influenza hemagglutinin). Lentiviral RhoJ short hairpin DNA was a kind gift from Dirk Geerts, AMC (CAACACTTGCTCGGACTGTATCTC).

### TIRF Microscopy

Cells were imaged with a Nikon Ti-E microscope equipped with a motorized TIRF Illuminator unit, a 60x TIRF objective (60x Plan Apo, Oil DIC N2, NA =1.49, WD = 120 um) and Perfect Focus System. Images were acquired with an Andor iXon 897 EMCCD camera and the Nikon NIS elements software. mNeonGreen was imaged using the 488 nm laser line and calcein red-orange was imaged using the 561 nm laser line. A quad split dichroic mirror (405 nm, 488 nm, 561 nm, 640 nm) was used in combination with dual band pass emission filter (515 to 545 nm, 600 to 650 nm). To achieve a larger field of view a 3 x 3 tile scans was acquired with 15% overlap stitching on the GFP channel. Time lapse images were taken every 10 s.

### Virus Production

Lentiviral particles were produced in HEK293T cells using 3rd generation packing plasmids. 2 and 3 days post transfection, supernatant was harvested and filtered (0.45 um) and concentrated using Lenti-X Concentrator (TaKaRa). Cells were selected with 1.5 ug/ml puromycin and used 2-6 days post transduction.

### SDS PAGE Gel and Western Blot

Cells were washed with PBS containing 1mM CaCl_2_ and 0.5 mM MgCl_2_, lysed in SDS-sample buffer (Life technologies) containing 4% β-mercaptoethanol (Sigma Aldrich) and denatured at 95 degrees for 10 minutes. Proteins were separated on a 4-12% gradient SDS-PAGE gel (Invitrogen) in MES buffer according to manufacturer’s protocol, and transferred to nitrocellulose membrane (Thermo Scientific Cat# 26619) in blot buffer (48 nM Tris, 39 nM Glycine, 0.04% SDS, 20% methanol). Membrane was blocked with 5% (w/v) milk (Campina) in Tris-buffered saline with Tween20 (TBST) for 60 minutes. The immunoblots were analyzed using primary antibodies incubated overnight at 4 degrees, washed three times with TBST, incubated with secondary antibodies linked to HRP (Dako, Aligent Technologies) and washed three times with TBST. HRP signals were visualized by enhanced chemiluminescence (ECL, cat# 32106, Thermo Scientific) for actin antibody and Super ECL (Thermo Scientific) for RhoJ antibody and light sensitive films (Fuji Film). Mouse monoclonal antibodies against RhoJ (cat# WH0057381M1) and actin (cat# A3853) were purchased from Sigma. Secondary HRP-conjugated goat anti-mouse (cat# P0447) antibody was purchased from Dako.

### Neutrophil Transendothelial Migration Under Physiological Flow

Blood used for neutrophil isolation was obtained from healthy adult volunteers. Whole blood with heparin was diluted (1:1) with 5% trisodium citrate in PBS, thereafter the diluted blood was pipetted on 12.5 ml Percoll (room temperature) 1.076 g/ml. The blood was centrifuged at 800g (room temperature) with slow start and brake for 20 minutes. The PBMC ring fraction and blood plasma were discarded and erythrocytes were lysed using ice cold lysis buffer (155 nM NH_4_CL, 10 mM KHCO, 0.1 mM EDTA). Thereafter, the neutrophils were washed with ice cold PBS. In between the neutrophils were centrifuged for 5 minutes at 450g at 4°C. The neutrophils were suspended in HEPES buffer (20 mM HEPES, 132 mM NaCl, 6 mM KCL, 1 mM CaCL_2_, 1 mM MgSO_4_, 1.2 mM K_2_HPO_4_, 5 mM D-glucose (Sigma-Aldrich), and 0.4% human serum albuman (Sanquin Reagents). Neutrophils were labelled with Calcein red-orange (Invitrogen) according to manufacturer’s protocol. Neutrophils were kept at room temperature for a maximum of 4 hours prior to experiment. HUVECs were cultured 48 before the experiment in an Ibidi 6 channel flow slide (µ-Slide VI 0.4, Cat.nr 80606, Ibidi) coated with fibronectin. HUVECs were stimulated with 10ng/ml TNFα (Peprotech) 4-20 hours prior to the experiment. Isolated neutrophils were activated at 37°C for 30 minutes, 1x10xxx2076 neutrophils were used per channel. A perfusion system was used to reach a flow of HEPES buffer of 0.5ml/minute (0.8 dyne/cm²) during the experiment, neutrophils were injected in the flow. The transendothelial migration was monitored for 20 minutes with a 10x air objective using a Zeiss Observer Z1 microscope. Live cell imaging was performed at 37°C with 5% CO_2_. The percentage of transmigrated neutrophils was calculated by dividing the number of neutrophils underneath the endothelium by the total neutrophil count. These percentages were calculated at 1, 2, 3, 4, 5, 10 and 15 minutes after the first neutrophils were in focus.

### Analyses

Images were analyzed using Fiji/ImageJ software. Images with labelled neutrophils were thresholded and analyzed using ‘Analyze Particles’. To quantify the tracks of the neutrophils Manual Tracking ImageJ plugin was used. A ‘turn’ was defined as a more than 90 degrees change in direction in the track of a specific neutrophil. To quantify focal adhesions, the images were thresholded and analyzed using ‘Analyze Particles’, including only particles with an area between 0.2 and 10 μm^2^. The speed of neutrophils underneath the endothelium was measured using MtrackJ, a plugin for Fiji. The neutrophils were tracked from the moment they were underneath the endothelium till the end of the experiment. The total length was divided through the time in seconds of the track. apical crawling was analyzed using Imaris software.

The crawling of neutrophils on top of the endothelium (apical) was measured using TrackMate, a plugin for Fiji, using spot finding, a blob diameter of 10.5 μm and spot threshold of 4000 or 300 depending on the imaging brightness/contrast. Simple LAP tracker was used with a minimal overlap of 10.5 μm, disabling gaps in tracks. All tracks that were not finished before the end of the movie (not finished crawling stage) or with less than 5 spots (to exclude rolling) were removed. Superplots were generated using the Super Plots Of Data web app (24).

## Results

### Neutrophils Change Shape Extensively During Basolateral Crawling

**Figure 1:**
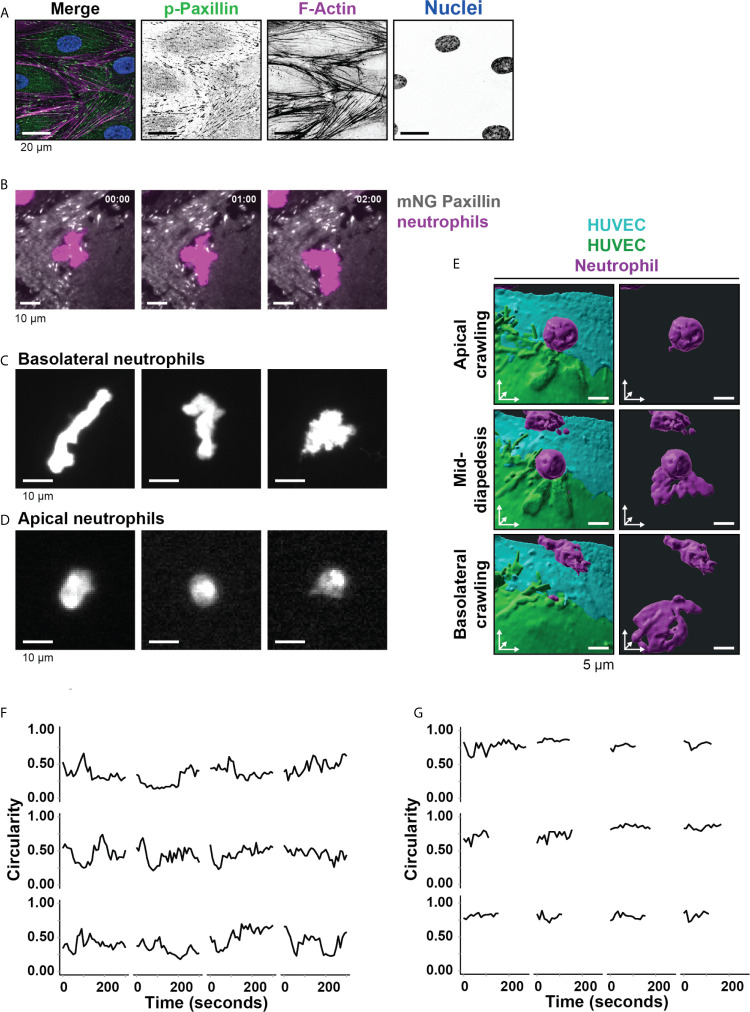
Neutrophil deformation at the basolateral side of the endothelium. **(A)** Immunofluorescent staining for phospho-paxillin (green), F-actin (magenta), and DNA (blue) on HUVECs stimulated for 20h with TNFα. Scale bar, 20 µm. **(B)** Stills from TIRF microscopy time lapse imaging showing neutrophils (magenta) and mNeonGreen-Paxillin (gray). Time indicated in seconds in upper right corner. Bar, 10 μm. **(C)** Stills from TIRF microcopy times lapse imaging showing 3 representative neutrophil shapes during crawling at the basolateral side of the endothelium. Bar, 10 μm. **(D)** Stills from widefield microscopy time lapse imaging showing 3 representative neutrophil shapes during crawling at the apical side of the endothelium. Bar, 10 μm. **(E)** 3D view stills from two ECs (green/turquoise) and a neutrophil (magenta) at three time points (apical crawling, mid-diapedesis, and basolateral crawling). Right panels show only the neutrophil. Bar, 5µm. **(F)** Quantification of the dynamics of circularity of neutrophils at the basolateral side (Basolateral). Each graph represents one neutrophil. **(G)** Quantification of the dynamics of circularity of neutrophils at the apical endothelial side (Apical). Each graph represents one neutrophil.

Endothelial cells are attached to the substratum through FA protein complexes (25). To examine the localization of FAs in cultured Human Umbilical Vein Endothelial Cells (HUVECs), we stained a monolayer of endothelial cells for F-actin and phospho-paxillin, a well-known marker for FAs (26). We observed phospho-paxillin puncta at the end of F-actin stress fibers (**Figure 1A**). Under inflammatory conditions, neutrophils cross the endothelium and stay under the endothelium for several minutes before continuing crossing the basement membrane and enter the underlying tissue (4). When monitoring this in real time using a transendothelial migration (TEM) flow model, neutrophils made several turns as if they were searching for a perfect route to prepare themselves to cross the basement membrane (**Supplementary Movie 1**).

From these observations, we hypothesized that FAs direct the basolateral route for neutrophils. To explore how neutrophils deal with FAs during TEM, we cultured mNeonGreen Paxillin-expressing HUVECs in specialized flow channels. The mNeonGreen-Paxillin is used as a proxy for FAs, enabling us to monitor in real-time FAs during neutrophil TEM under physiological flow conditions. By using TIRF microscopy, we specifically detected the basolateral side of the endothelial cell layer and were able to detect the FAs with high contrast in real-time. When studying both FA and neutrophil behavior at the basolateral side, it seems that neutrophils were trapped by surrounding FAs as if they experienced a physical barrier (**Figure 1B** and **Supplementary Movie 2**). We found that the basement membrane proteins laminin, collagen and fibronectin were deposited by the HUVEC upon culturing (**Supplementary Figure 1A**). These proteins can form a confined surrounding that potentially can be used by neutrophils to migrate underneath the endothelium. For the neutrophils crawling at the basolateral side, we observed a wide variety of neutrophil shapes, ranging from stretched out to bended (**Figure 1C**, **Supplementary Figure 1B** and **Supplementary Movie 3**). These morphological changes were specific for neutrophils at the basolateral side, as we did not observe these changes for neutrophils that were crawling at the apical side of the endothelium prior to crossing the endothelium (**Figure 1D** and **Supplementary Figure 1C**). To illustrate these morphological changes in more detail, we used 3D lattice light sheet imaging (**Figure 1E** and **Supplementary Figure 1D**). These recordings showed the morphological shape transition neutrophils undergo as soon as they cross the endothelium. To measure these morphological changes, neutrophil circularity during basolateral and apical crawling was determined using time-lapse recordings. Circularity was measured by 4π(area/perimeter^2^) with 1 being perfectly round. Apical crawling neutrophils showed a circularity of 0.74 (**Figure 1F**), whereas basolateral crawling neutrophils showed a circularity of 0.43 (**Figure 1G**). Thus, neutrophils that crawled underneath the endothelium showed a higher degree of deformation than neutrophils that crawled on the apical surface of the endothelium.

**Figure 2:**
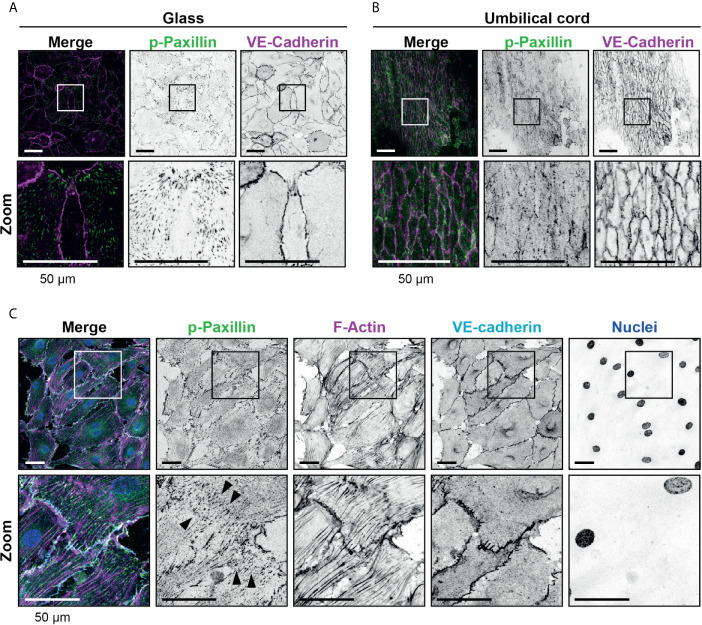
Presence of focal adhesions at endothelium from the umbilical cord. **(A)** Immunofluorescent staining for phospho-paxillin (green) and VE-cadherin (magenta) of HUVECs grown on glass substrate. ROI is indicated in the merge. Images are inverted for clarification. Lower panel shows zoom from ROI. Scale bar, 50 µm. **(B)** Immunofluorescent staining for phospho-paxillin (green) and VE-cadherin (magenta) of HUVECs on an umbilical cord. ROI is indicated in the merge. Images are inverted for clarification. Lower panel shows zoom from ROI. Scale bar, 50 µm **(C)** Immunofluorescent staining for phospho-paxillin (green), F-actin (magenta), VE-cadherin (cyan) and nuclei (blue) of BOEC seeded on umbilical cord, treated with TNF-alpha. ROI is indicated in the merge. Images are inverted for clarification. Lower panel shows zoom from ROI. Black arrowheads indicate focal adhesions. Scale bar, 50 µm.

The endothelial cells used are cultured on glass cover slips to allow TIRF microscopy. It is recognized that stiff surfaces can influence structures like FAs (27). To study FAs in endothelial cells on more softer substrates, we used umbilical cord tissue and replated HUVECs onto the umbilical cord substrate that were depleted from endothelial cells first. These experiments showed the presence of FAs in endothelial cells *in vivo* albeit much more at the junctional region. Although endothelial cells of the umbilical cord were smaller in size, the presence and number of FAs per surface area was comparable with what was observed for *in vitro* cultures (**Figures 2A, B** and **Supplementary Figures 1E, F**). These data are in line with previous reports showing the presence of FAs in different vascular beds (8). Additional treatment of the endothelial cells in umbilical cords with TNFα showed similar numbers of FAs compared to the *in vitro* setup, namely 0.05 ( ± 0.01, standard deviation) per μm^2^ and an average size of 0.75 ( ± 0.04, standard deviation) μm^2^ and an increase in FAs and stress fibers across the axis of the endothelial cell (**Figure 2C**). FAs have been recognized *in vivo* as well, albeit less prominent (8). To verify FAs under more *in vivo* conditions, we stained human mesenteric vessels and showed the presence of FAs in the endothelium (**Supplementary Figure 2A**).

### Neutrophils Navigate Around Focal Adhesions

**Figure 3:**
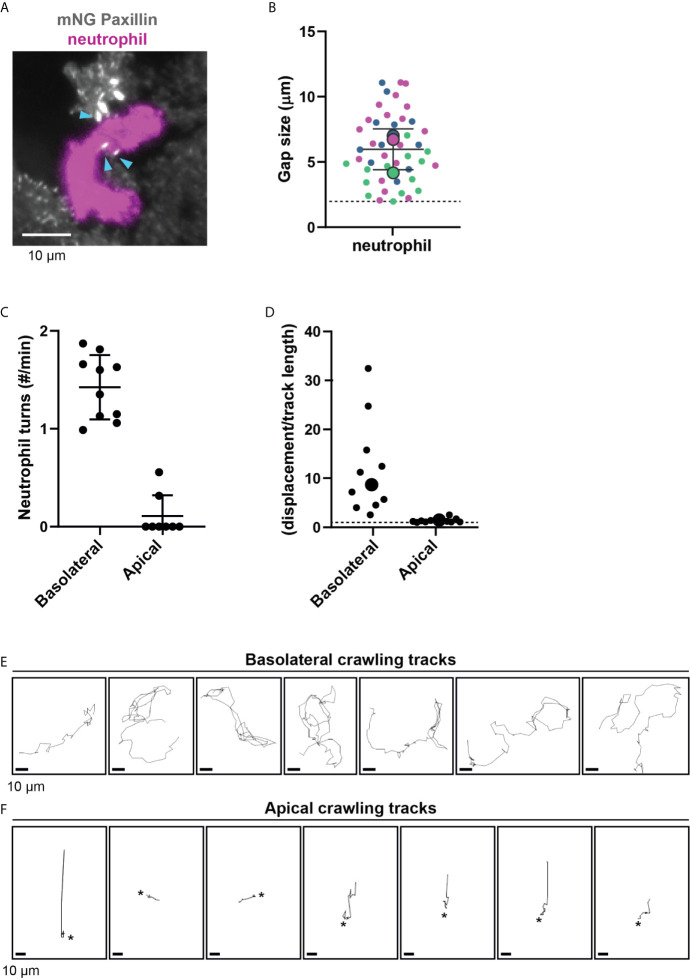
Neutrophil crawling at the apical and basolateral side of the endothelium. **(A)** Still from TIRF microscopy time lapse imaging showing neutrophil (magenta) and mNeonGreen-Paxillin (gray). Neutrophil squeezes through a small gap between three focal adhesions indicated with blue arrowheads. Bar, 10 μm. **(B)** Quantification of gap size between focal adhesions where neutrophils can navigate through. Superplot in which small dots are individual datapoints, large dots represent means from 3 independent experiments (magenta, green, blue). Mean and standard deviation are indicated. Dotted line at 2 µm, indicating the minimum distance between two focal adhesions neutrophils can still migrate through. **(C)** Quantification of the amount of turns (>90˚) basolateral and apical neutrophils make per minute. Data from three independent experiments. Each dot represents an individual neutrophil. **(D)** Superplot of ratios of the effective displacement (distance between beginning and end of the migratory track) and the total displacement (length of the migratory track) of basolateral and apical neutrophils. Each dot represents an individual neutrophil. Dotted line represents 1, meaning straight migration paths. Large dots represent means. Welch’s T-test p=0.03951772 **(E)** Crawling tracks of neutrophils at the basolateral side of the endothelium. Each frame is about 11 seconds. Bar, 10 μm. **(F)** Crawling tracks of neutrophils at the apical side of the endothelium. Each frame is about 11 seconds. Asterisks indicate start of diapedesis. Bar, 10 μm.

We hypothesized that neutrophil deformation is imposed by FAs in the subendothelial space. Indeed, neutrophils changed shape to squeeze between FAs (**Figure 3A** and **Supplementary Movie 4**). We quantified the minimum distance between two FAs where a neutrophil can still pass through. This was on average 6 μm, but the minimal distance between two adhesions was as small as 2 μm (**Figure 3B**), indicating that neutrophils underneath the endothelium drastically deform to crawl themselves in between individual FAs.

In addition, neutrophils that crawled at the basolateral level frequently changed their migration direction. To quantify this, we tracked neutrophil migration paths and defined a change in direction as a “turn” when an angle in the migration path was more than 90°. One individual neutrophil was able to change its direction multiple times per minute. We did not see this effect for neutrophils that crawled on the apical surface of the endothelium, as they only occasionally change direction (**Figure 3C**). This suggests that FAs may indeed act as physical obstacles for neutrophils. Related to this finding, we noticed that the basolateral crawling speed of neutrophils is remarkably high, but their net displacement is rather limited (**Figure 3D**). In contrast, the net displacement of apical neutrophils was similar to their total displacement or track length, indicated by a ratio of 1 (**Figure 3D**). By studying the individual crawling tracks, the motion did not occur as directional but rather random (**Figure 3E**). This is in sharp contrast to neutrophils that crawled at the apical side of the endothelium, where migration tracks were often more directional (**Figure 3F**). Together, the findings on the difference of neutrophil crawling either on the apical or basolateral side of the endothelium support the notion that FAs act as physical obstacles for neutrophil basolateral migration.

### Neutrophils Navigate Around Focal Adhesions During Basolateral Crawling

**Figure 4:**
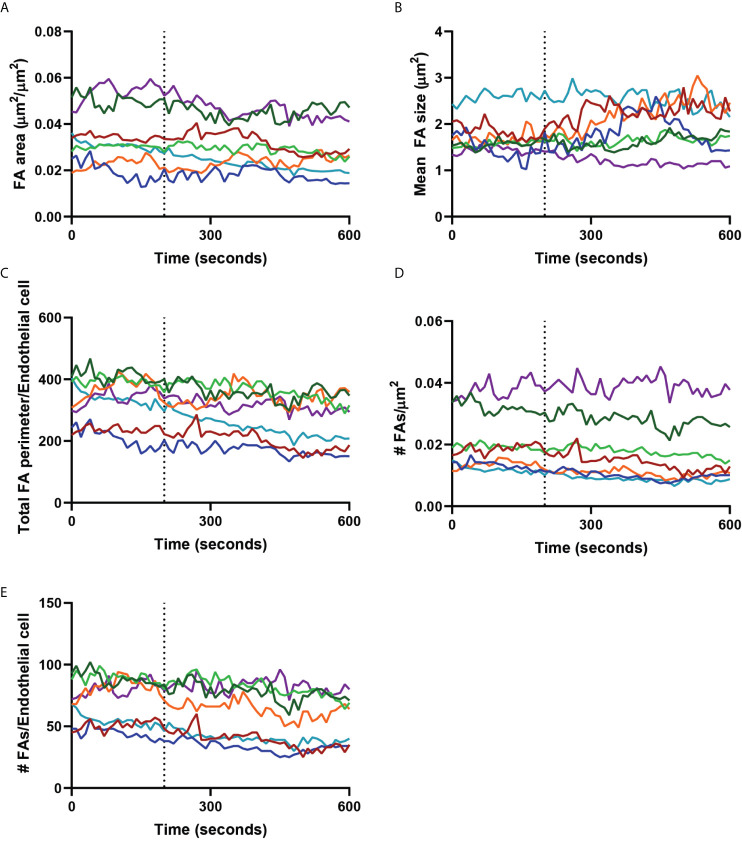
Focal adhesions during neutrophil crawling. Quantification of the focal adhesions (FA) of an endothelial cell with a neutrophil crawling underneath from the dotted line (diapedesis) onwards. Colored lines indicate seven representative neutrophils. **(A)** Graph shows the total focal adhesion area per µm^2^. **(B)** Graph shows the mean FA size. **(C)** Graph shows the total perimeter of all the focal adhesions of one endothelial cell. This is a measure for the shape of the focal adhesions. **(D)** Graph shows the number of FAs per µm^2^. **(E)** Graph shows the number of FAs per endothelial cell.

As we observed that neutrophils navigated around FAs, we hypothesized that neutrophils did not disrupt or change the turnover of FAs. To test this, we measured the area and perimeter of individual endothelial FAs before and during basolateral crawling of neutrophils. To quantify FA stability, we calculated the total FA area of a region of interest containing one endothelial cell including a neutrophil that was crawling at the basolateral side. We compared this area before neutrophil arrival at the subendothelial space to the total area after the neutrophil migrated underneath the endothelial cell. This analysis showed that the total FA area did not change upon basolateral crawling of neutrophils (**Figure 4A**). Additionally, the average size of FAs did not change during basolateral crawling of neutrophils (**Figure 4B**). To determine if basolateral crawling of neutrophils changed the shape of FAs, we determined the total perimeter of all FAs in the field of view. Again, we did not find any difference of the FA perimeter upon basolateral crawling of neutrophils (**Figure 4C**). Furthermore, the number of focal adhesions did not change (**Figures 4D**, **E**). Thus, neutrophil TEM including basolateral crawling is a non-destructive process that retains FA integrity. This suggests that FA density can act as barrier for neutrophil migration at the basolateral side.

### RhoJ Depletion Results in Reduced Basolateral Crawling Velocity

**Figure 5:**
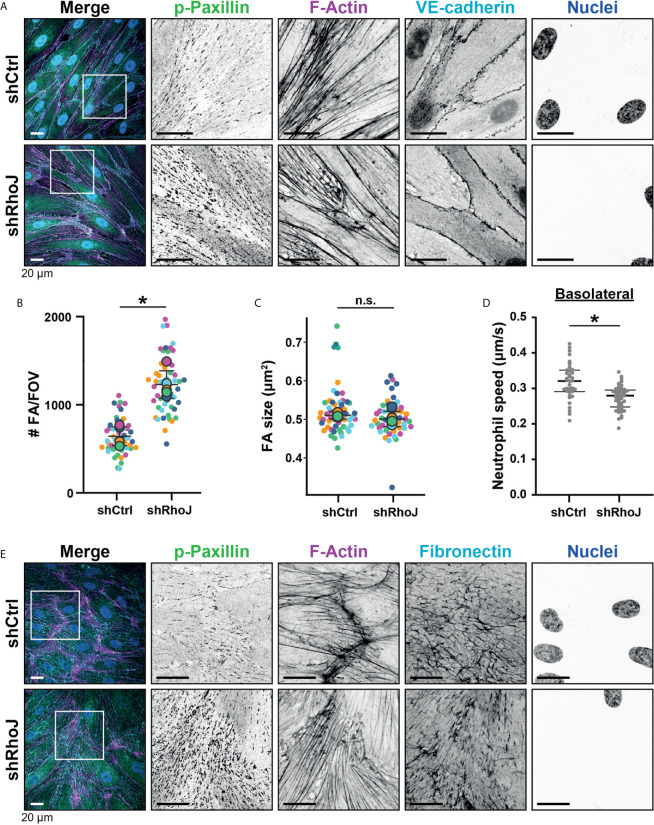
Focal adhesions upon RhoJ silencing. **(A)** Immunofluorescent staining for phospho-paxillin (green), F-actin (magenta), VE-cadherin (Turquoise) and DNA (blue) on HUVECs stimulated for 20h with TNFα. ROI is indicated in the merge. Images are inverted for clarification. Upper panel are control cells (shCtrl), lower panel are cells with RhoJ depletion (shRhoJ). Scale bar, 20 µm. **(B)** Quantification of the number of focal adhesions (FA) per field of view (FOV) for control (shCtrl) and RhoJ knockdown (shRhoJ) conditions. Superplot in which small dots are individual datapoints, large dots represent means from 5 independent experiments (five colors). Mean and standard deviation are indicated. Welch’s T-test *p=0.000218. **(C)** Quantification of the size of focal adhesions (FA) for control (shCtrl) and RhoJ knockdown (shRhoJ) conditions. Superplot in which small dots are individual datapoints, large dots represent means from 5 independent experiments (five colors). Mean and standard deviation are indicated. Welch’s T-test p=0.2712415, not significant (n.s.). **(D)** Quantification of the speed of basolateral neutrophils under control (shCtrl) and RhoJ knockdown (shRhoJ) endothelial cells. Violin plot in which small dots are individual datapoints. Mann-Whitney U-test: p<0.05. **(E)** Immunofluorescent staining for phospho-paxillin (green), F-actin (magenta), Fibronectin (Turquoise) and DNA (blue) on HUVECs stimulated for 20h with TNFα. ROI is indicated in the merge. Images are inverted for clarification. Upper panel are control cells (shCtrl), lower panel are cells with RhoJ depletion (shRhoJ). Scale bar, 20 µm.

The small GTPase RhoJ regulates FA turnover, thereby dictating FA density (11). To study if an increase in FA density reduced neutrophil basolateral crawling, we depleted RhoJ from endothelial cells. RhoJ was silenced with an efficacy of >90% (**Supplementary Figures 2B, C**), which resulted in an almost 2-fold increase in the number of FAs (**Figures 5A**, **B**), with no change in FA size (**Figure 5C**). These results enabled us to study if increasing number of FAs perturbed neutrophil TEM and basolateral migration. Indeed, RhoJ silencing reduced neutrophil motility during basolateral crawling (**Figure 5D**), suggesting that increased number of FAs resulted in more obstacles for neutrophils to overcome when migrating underneath the endothelium. To rule out an effect of the underlying matrix that is deposited by the endothelial cells, we performed immunofluorescence staining of the matrix protein fibronectin, known to be involved in inflammation (28). These data showed no difference in basolateral deposition of fibronectin upon RhoJ depletion (**Figure 5E**). From these experiments we concluded that increasing the number of FAs decreased basolateral crawling speed of neutrophils.

### Endothelial RhoJ Depletion Does Not Affect Apical Crawling and Diapedesis of Neutrophils

**Figure 6:**
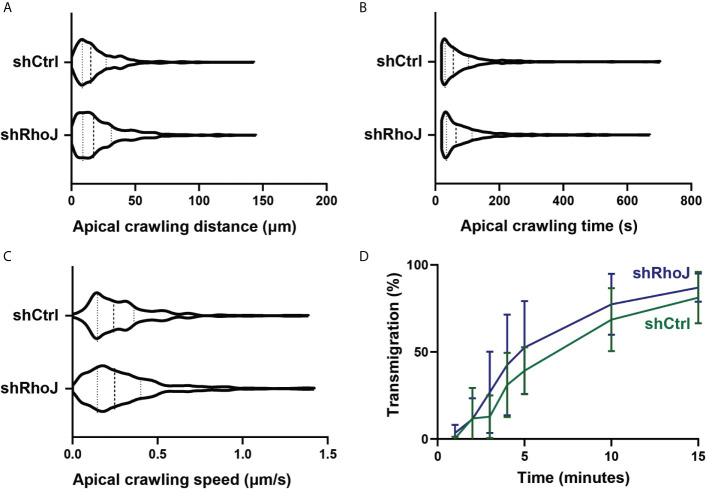
Transendothelial migration upon RhoJ silencing in endothelial cells. **(A)** Quantification of the distance of apical neutrophils in the crawling stage on control (shCtrl) and RhoJ knockdown (shRhoJ) endothelial cells. Violin plot of three independent experiments including at least 100 neutrophils per experiment **(B)** Quantification of the apical crawling time of neutrophils on control (shCtrl) and RhoJ knockdown (shRhoJ) endothelial cells. Violin plot of three independent experiments including at least 100 neutrophils per experiment. **(C)** Quantification of the speed of apical neutrophils in the crawling stage on control (shCtrl) and RhoJ knockdown (shRhoJ) endothelial cells. Violin plot of three independent experiments including at least 100 neutrophils per experiment. **(D)** Quantification of the percentage of neutrophils that underwent diapedesis for control endothelial cells (shCtrl, green) and RhoJ silenced endothelial cells (shRhoJ, blue). Data from three independent experiments including at least 100 neutrophils per experiment. Error bars show standard deviation.

To study if the number of FAs regulate TEM efficiency, we focused on the different steps of the TEM process under flow conditions. To increase the number of FAs, we used endothelial cells that were silenced for RhoJ or treated with shCtrl. For the apical crawling distance, we did not find any difference of neutrophil crawling between RhoJ-depleted and control endothelial cells (**Figure 6A**). Also, the crawling time, i.e., the time from crawling until diapedesis as well as the speed, did not differ between both endothelial cell treatments (**Figures 6B**, **C**). In line with these findings, RhoJ-deficient endothelial cells did not change the number of neutrophils that transmigrated (**Figure 6D**). This suggests that increasing the number of FAs by RhoJ depletion did not affect neutrophil TEM efficiency.

### TIAM-1 Increases FA Number and Blocks Basolateral Crawling of Neutrophils

**Figure 7:**
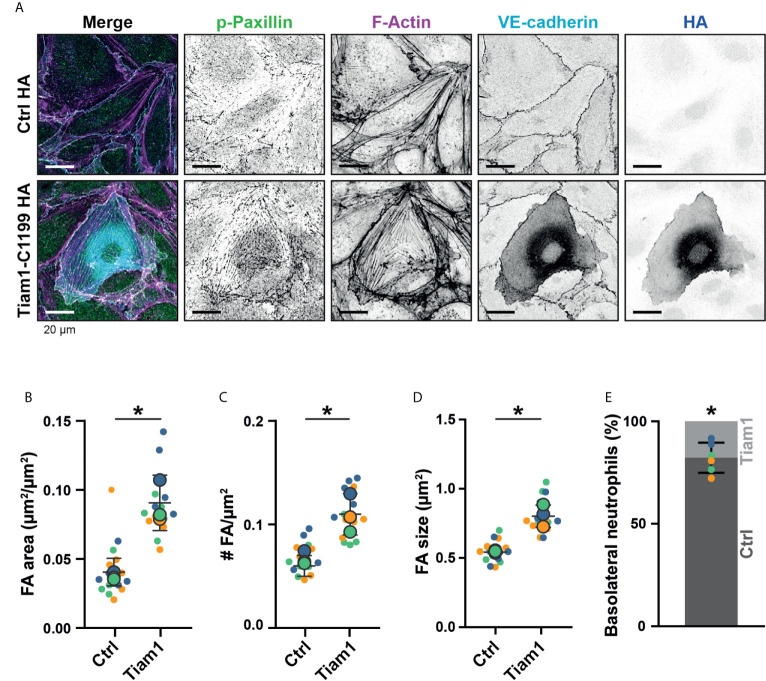
Focal adhesions upon Tiam1-C1199 expression. **(A)** Immunofluorescent staining for phospho-paxillin (green), F-actin (magenta), VE-cadherin (Turquoise) and HA (blue) on HUVECs stimulated for 20h with TNFα. Images are inverted for clarification. Upper panel are control cells (Ctrl HA), lower panel are cells expressing Tiam1-C1199 (Tiam1-C1199). Scale bar, 20 µm. **(B)** Quantification of the area focal adhesions (FA) cover or control (Ctrl) and Tiam1-C1199-expressing cells (Tiam1-C1199). Superplot in which small dots are individual datapoints, large dots represent means from 3 independent experiments (three colors). Mean and standard deviation are indicated. Welch’s T-test *p=0.02411431. **(C)** Quantification of the number of focal adhesions (FA) per field of view (FOV) for control (Ctrl) and Tiam1-C1199-expressing cells (Tiam1-C1199). Superplot in which small dots are individual datapoints, large dots represent means from 3 independent experiments (three colors). Mean and standard deviation are indicated. Welch’s T-test *p=0.04694375. **(D)** Quantification of the size of focal adhesions (FA) in control (Ctrl) and Tiam1-C1199-expressing cells (Tiam1-C1199). Superplot in which small dots are individual datapoints, large dots represent means from 3 independent experiments (three colors). Mean and standard deviation are indicated. Welch’s T-test *p=0.02819648 **(E)** Quantification of the percentage of TEM events in which a neutrophil migrates at a control-Tiam1-C1199 junction and migrates further underneath the Tiam1-C1199 cell (light bar) versus neutrophils that migrate further underneath the control cell (dark bar). Dots represent individual datapoints from three experiments (three colors). Error bar shows 95% confidence interval. One-sample Wilcoxon test: p<0.05.

We hypothesized that if the number of FAs at junction regions can be specifically increased, we may be able to steer neutrophil basolateral migration at the junction regions. By increasing the number of FA in one endothelial cell to the level that neutrophils cannot pass through, but not in the adjacent one, neutrophils would migrate underneath the endothelial cell that allow neutrophils to squeeze through FAs. Overexpression of an active mutant of the GEF Tiam1, Tiam1-C1199, induced massive numbers of FAs (**Figure 7A** and **Supplementary Figure 2D**) (29). Additionally, we found VE-cadherin to be more diffusely distributed over the cell surface. The total area that FAs covered was more than doubled (**Figure 7B**). Furthermore, the number of FAs was elevated (**Figure 7C**) and their size was increased (**Figure 7D**). By transiently expressing Tiam1-C1199, we obtained a monolayer of HUVECs with a mix of normal FA number, i.e., non-transfected endothelial cells and Tiam1-positive endothelial cells showing increased number of FAs, in particular at junction regions. We used this system to study whether neutrophils preferred either of the two cell types for basolateral crawling. The number of neutrophils that crossed the endothelium was unaltered. However, we observed that crawling neutrophils seem to avoid Tiam-1-expressing cells. Upon diapedesis, we found that neutrophils first started to penetrate underneath the Tiam1-C1199-expressing endothelial cell but after 2-3 µm underneath this cell, the neutrophil turned and migrated away from the Tiam1-positive endothelial cell and continued migrating underneath the control endothelial cell (**Figure 7E**, **Supplementary Figure 3A** and **Supplementary Movie 5**). These results clearly demonstrated that neutrophils preferred endothelial cells with lower numbers of FAs for basolateral crawling. Moreover, we observed that neutrophils that transmigrated at cell-cell junctions, consisting of two Tiam1-expressing endothelial cells, were trapped at the junction (**Supplementary Figure 3B** and **Supplementary Movie 6**). This observation suggest that neutrophils can be hindered at the basolateral level by increasing the number of FAs that function as physical obstacles for neutrophils. To study if potential other mechanisms can drive preferential basolateral migration of neutrophils under neath control endothelial cells, like decreased adhesion molecule expression at the Tiam1-C1199 cells compared to the control endothelial cells, we performed immunofluorescent stainings. By using TIRF microscopy, we specifically imaged the basolateral side of the endothelium and found that the expression levels of ICAM-1 in Tiam1-C1199 cells were comparable to control cells (**Supplementary Figures 3C, D**). Moreover, also PECAM-1 stainings showed a similar pattern between control and Tai1-C1199 expressing endothelial cells (**Supplementary Figure 3E**). It should be noted that endothelial cells that express Tiam1-C1199 showed a larger morphology, most likely due to intrinsic rac1 activation, a well-known regulator of endothelial cell size (29). The percentage of neutrophils that underwent diapedesis across Tiam1-C1199-expressing HUVEC was similar to control situations.

Together, our data suggest that endothelial FAs can mark the basolateral migration path for neutrophils and may thereby regulate efficient entering of the underlying tissue.

## Discussion

Although many studies focus on the apical crawling of leukocytes that initiates the TEM process, little is known about leukocyte migration underneath the endothelium once they have crossed it. It is well recognized that neutrophils reside for about 20 minutes at the subendothelial space (4), indicating the importance of this phase. It is believed that they search for a spot to escape the basement membrane and the pericyte layer to enter the underlying tissue (30). However, it is unclear what factors contribute to the pause before the tissue is invaded. To increase our understanding of factors that regulate neutrophil migration at the basolateral level of the endothelium, we focused on this post-diapedesis step of the inflammatory response using TIRF microscopy.

Underneath the endothelium, neutrophils encounter focal, protein complexes that link the endothelial cell to the underlying substratum (31). Interestingly, when observing neutrophils migrating at this basolateral side of the endothelium, it appears that the endothelial cells themselves are not hampered in any way by the presence of these neutrophils. Endothelial cells remain firmly bound to the substratum. Using TIRF microcopy, our data show that neutrophils leave FAs intact and migrate around these static structures rather than breaking them down. Our observations contrast a previous study on T-cells in which FAs were studied with interference reflection microscopy, which is a label-free method. In that study, detachment was observed and it was proposed that transient weakening of FA attachment was required to widen subendothelial spaces (9). However, the signal of the T-cells interfered with that of the FAs and therefore unambiguous identification of FAs was not possible. Here, using dual color fluorescence imaging we were able to identify both FAs and neutrophils with high spatial and temporal resolution. Our observations support the idea that FAs at the basolateral side of the endothelium remain intact and act as physical obstacles.

Our data shows that the minimum gap between FAs through which neutrophils could squeeze themselves was 2 µm. During the migration through these gaps, neutrophils drastically deform their cell body. It was previously reported that using a system with artificial pores, neutrophils prefer pores sizes of at least 2 µm (32). This cut-off is strikingly similar to the size limit that we found for neutrophils, suggesting that the cellular deformation is triggered by physical cues rather than a chemical or signaling factor.

The largest organelle of the neutrophil, the nucleus, is multi-lobular, making it easier to deform. It is known that the nucleus is particularly important during migration of neutrophils. Renkawitz and colleagues showed that the nucleus can be used as a mechanical gauge that neutrophils use to migrate through a narrow pore (32). Similar to what we observed in our experiments, protrusions from the neutrophils poked into several pores, but once the nucleus fit through a certain narrow pore, the other protrusions are retracted, and the neutrophil migrated forward (32). This notion of the neutrophil trying several routes before choosing the path of least resistance and squeezing through two individual FAs was supported by our observations. These observations are also in line with the work by Barzilai and co-workers (33). They showed that the neutrophil used its nucleus as a “drill” to initiate the diapedesis step and penetrate the endothelium (33). Our data now add to the idea that the nucleus is not only used to penetrate the endothelial monolayer but is also used to find its way through the physical obstacles made by the basolateral FAs.

Our work indicates that FAs act as obstacles for neutrophils. It is important to note that FAs also include the basement membrane protein underneath the FA. Therefore, it is possible that the basement membrane itself may also function as an obstacle for migrating neutrophils. Interestingly, we noticed reduced presence of basement membrane proteins underneath the nucleus. This may explain why we see not so many neutrophils crawling underneath the nucleus. In particular under inflammatory conditions, the composition of the extracellular matrix can change, having an effect on how leukocytes may cross the vascular border (REF). In fact, different vascular beds display different basement membrane proteins (REF). And as the composition of the subendothelial space plays an important role in TEM, it is an important factor to consider when studying leukocyte transmigration. This consideration is often not included in *in vitro* studies. The matrix underneath the endothelium is far from homogenous. So-called low expression regions (LERs) have been identified by Wang and colleagues as sites with considerably lower expression of key vascular basement membrane constituents (34). Additional work by the Sorokin lab showed that basal membrane proteins such as laminin 511 can act as barriers for neutrophils to cross the endothelial cell junctions (35). They furthermore show in additional work that loss of endothelial laminin 511 results in enhanced experimental autoimmune encephalomyelitis due to increased T cell infiltration (36). These LERs are associated with gaps between pericytes and are preferentially used by transmigrating neutrophils (4). Neutrophils seem to prefer the path of least resistance, as supported by our experiments that showed that increased number of FAs prevents proper basolateral crawling of neutrophils. Interestingly, the gap size through which neutrophils crossed the pericyte layer and the basement membrane was entirely dependent on the presence of neutrophils and appeared to involve neutrophil-derived serine proteases (34). Wang and colleagues showed neutrophil-dependent degradation of basement membrane proteins, whereas our study among others (9) show that the size and number of FAs are not changed in the presence of neutrophils. This suggests that neutrophils have two ways of migrating through confined spaces: 1. By navigating around physical obstacles and 2. By taking the path of least resistance and degrade matrix proteins. In this way neutrophils could efficiently migrate into the tissue without causing unnecessary tissue damage.

In the present study, we did not take the role of pericytes into account. However, it is clear that pericytes play a particularly important role in the extravasation of neutrophils *in vivo* (4). Neutrophils use their integrins to bind to ICAM-1 on pericytes and migrate towards gaps in the pericyte layer. These gaps are enlarged upon inflammation because of shape changes of pericytes, making space for neutrophils to exit the vessel wall (4). Neutrophils exhibit abluminal crawling for about 20 minutes before migrating further into the tissue. In this time, they migrated about 54 µm between the endothelial cells and pericytes (4). This underscores the importance of this post-diapedesis stage of TEM. Using our simplified setup without pericytes and with the use of the TIRF microscopy, we could specifically focus on FAs only.

Furthermore, substrate stiffness of the underlying tissue might also play an important role (37). We previously showed that substrate stiffness is translated by the endothelium *via* the protein DLC-1. This mechanism mainly affects the adhesion stage but not the diapedesis itself (37). Another study confirmed these findings and showed that neutrophil TEM increased with increasing substrate stiffness (38). However, extracellular matrix rigidity does induce FA formation in both arterial and venous endothelial cells (8). Therefore, it may also affect basolateral crawling, and this might result in altered inflammatory response.

In summary, we show that endothelial FAs act as physical obstacles for neutrophils that crawl at a basolateral side of the endothelium. FAs cause deformation of the neutrophil and slow down or even block basolateral crawling. This in the end may determine the efficiency for neutrophils entering the inflamed tissue and reaching the place of infection.

## Data Availability Statement

The raw data supporting the conclusions of this article will be made available by the authors, without undue reservation.

## Author Contributions

JA, EM, LS, MG, SN, BK, MF-B, JR, DG, and SH: Data collection, analysis, and interpretation. JB and JG: Project design. JA, JB, and JG: Drafting and writing the manuscript. All authors contributed to the article and approved the submitted version.

## Funding

This work was supported by ZonMW NWO Vici grant # 91819632 (JB/MG) and NWO ALW-OPEN grant ALWOP.306 (EM).

## Conflict of Interest

The authors declare that the research was conducted in the absence of any commercial or financial relationships that could be construed as a potential conflict of interest.

## Supplementary Material

Supplementary Figure 1
**(A)** Immunofluorescent staining for Laminin α4, Laminin 1/2, Collagen IV, or Fibronectin (green) and VE-cadherin (magenta). Right panel shows the merged image. Scale bar, 20 µm. **(B)** Stills from TIRF microcopy times lapse imaging showing 3 representative neutrophil shapes during crawling at the basolateral side of the endothelium. Bar, 10 μm. **(C)** Stills from widefield microscopy time lapse imaging showing 3 representative neutrophil shapes during crawling at the apical side of the endothelium. Bar, 10 μm. **(D)** 3D view stills from two ECs (green/turquoise) and a neutrophil (magenta) at three time points (apical crawling, mid-diapedesis, and basolateral crawling). Right panels show only the neutrophil. Bar, 5µm. **(E)** Immunofluorescent staining for phospho-paxillin (green) and VE-cadherin (magenta) of HUVECs grown on glass substrate. ROI is indicated in the merge. Images are inverted for clarification. Lower panel shows zoom from ROI. Scale bar, 50 µm. **(F)** Immunofluorescent staining for phospho-paxillin (green) and VE-cadherin (magenta) of HUVECs on an umbilical cord. ROI is indicated in the merge. Images are inverted for clarification. Lower panel shows zoom from ROI. Scale bar, 50 µm.

Supplementary Figure 2
**(A)** Immunofluorescent staining of mesenteric artery for phospho-paxillin (green), F-actin (magenta) and VE-cadherin (cyan). Lower panels are zoom of the ROI indicated in upper panels. Scale bar, 20 µm. **(B)** Western blot analysis shows successful knock down of RhoJ. Actin is shown as loading control. **(C)** Quantification of Western blot in (A). Error bar median with 95% confidence interval. Mann-Whitney U-test: p<0.05. **(D)** Immunofluorescent staining for phospho-paxillin (green), F-actin (magenta), VE-cadherin (Turquoise) and HA (blue) on HUVECs stimulated for 20h with TNFα. ROI is indicated in the merge. Images are inverted for clarification. Upper panel are control cells (Ctrl HA), lower panel are cells expressing Tiam1-C1199 (Tiam1-C1199). Scale bar, 20 µm.

Supplementary Figure 3
**(A)** DIC stills from time lapse image showing three Tiam1-C1199 cells (dotted white line) and neutrophils that transmigrate at a ctrl-Tiam1-C1199 junction. Basolateral crawling tracks of these neutrophils are indicated in blue. Stills from Supplemental Movie 5. Time indicated in seconds in upper right corner. Bar, 50 μm. **(B)** DIC stills from time lapse image showing three Tiam1-C1199 cells (dotted white line) and a neutrophil that transmigrates at a ctrl-Tiam1-C1199 junction (arrowhead). The neutrophil remains stuck between the two Tiam1-C1199 cells. Stills from Supplemental Movie 6. Time indicated in seconds in upper right corner. Bar, 50 μm. **(C)** Immunofluorescent staining for ICAM-1 (green) and HA (Tiam1-C1199 HA) (magenta) on HUVECs stimulated for 4h with TNFα. ROI is indicated in the merge. Images are acquired using TIRF microscopy. Scale bar, 20 µm. **(D)** Quantification of ICAM-1 expression levels using MFI (mean fluorescence intensity) in HUVEC expressing Tiam1-C1199 or control (ctrl), all stimulated for 4h with TNFα. Lines indicate medians. **(E)** Immunofluorescent staining for PECAM (green) and HA (Tiam1-C1199 HA) (magenta) on HUVECs stimulated for 4h with TNFα. ROI is indicated in the merge. Images are acquired using confocal microscopy, maximum intensity projection. Scale bar, 20 µm. **(F)** Table with statistical information of the superplots (**Figures 3D**, **5B**, **5C**, **7B**, **7C**, **7D**).

Supplementary Movie 1 DIC time lapse imaging of TEM followed by basolateral crawling, showing extensive change of direction. Time is indicated in seconds in upper left corner. Bar, 20 μm.

Supplementary Movie 2 Time lapse TIRF imaging of basolateral crawling of neutrophils, showing that focal adhesions act as physical barriers for neutrophils. mNeonGreen-Paxillin (grey) expressing endothelial cells and labelled neutrophils (magenta). Time is indicated in seconds in upper left corner. Bar, 20 μm.

Supplementary Movie 3 Time lapse TIRF imaging of basolateral crawling of neutrophils, showing dynamic changes in cell shape. Endothelial cells (not shown) and labelled neutrophils (grey). Time is indicated in seconds in upper left corner. Bar, 20 μm.

Supplementary Movie 4 Time lapse TIRF imaging of basolateral crawling of neutrophils, showing that focal adhesions act as physical barriers for neutrophils. mNeonGreen-Paxillin (grey) expressing endothelial cells and labelled neutrophils (magenta). Time is indicated in seconds in upper left corner. Bar, 20 μm.

Supplementary Movie 5 DIC time lapse image of three Tiam1-C1199 cells (center) and neutrophils that avoid crawling underneath the Tiam1-C1199 cells. Stills are depicted in supplemental **Figure 2B**. Time is indicated in seconds in upper left corner. Bar, 50 μm.

Supplementary Movie 6 DIC time lapse image of two Tiam1-C1199 cells (center) and a neutrophil that is trapped at the junction in between these two Tiam1-C1199 cells. Stills are depicted in supplemental **Figure 2C**. Time is indicated in seconds in upper left corner. Bar, 50 μm.
