# Supplementary figures and images for: Endothelial Focal Adhesions Are Functional Obstacles for Leukocytes During Basolateral Crawling
Source: Front Immunol. 2021 May 18;12:667213. doi: 10.3389/fimmu.2021.667213 (PMC8167051; doi:10.3389/fimmu.2021.667213)

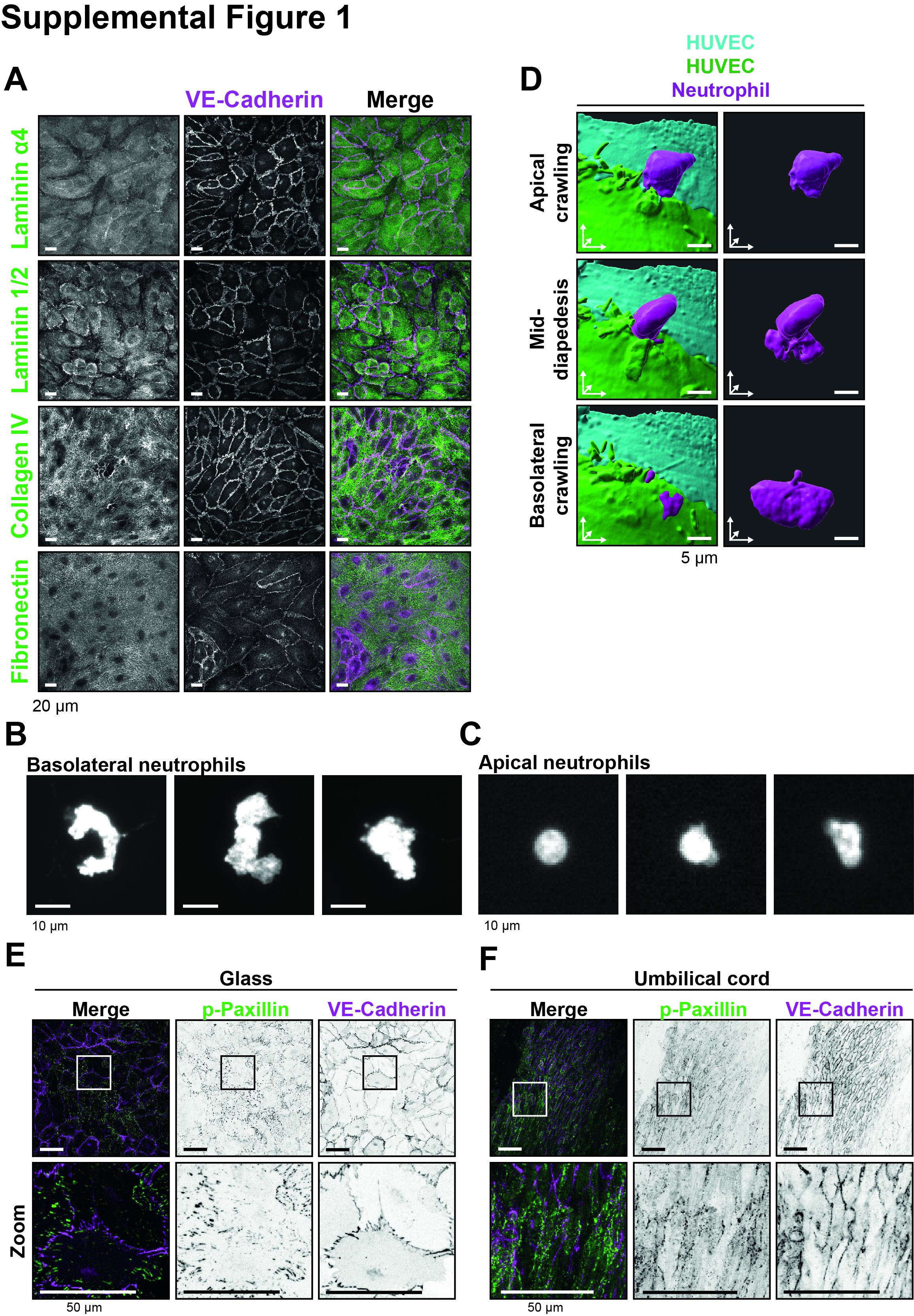

Supplement: Supplementary Figure 1 — (A) Immunofluorescent staining for Laminin α4, Laminin 1/2, Collagen IV, or Fibronectin (green) and VE-cadherin (magenta). Right panel shows the merged image. Scale bar, 20 µm. (B) Stills from TIRF microcopy times lapse imaging showing 3 representative neutrophil shapes during crawling at the basolateral side of the endothelium. Bar, 10 μm. (C) Stills from widefield microscopy time lapse imaging showing 3 representative neutrophil shapes during crawling at the apical side of the endothelium. Bar, 10 μm. (D) 3D view stills from two ECs (green/turquoise) and a neutrophil (magenta) at three time points (apical crawling, mid-diapedesis, and basolateral crawling). Right panels show only the neutrophil. Bar, 5µm. (E) Immunofluorescent staining for phospho-paxillin (green) and VE-cadherin (magenta) of HUVECs grown on glass substrate. ROI is indicated in the merge. Images are inverted for clarification. Lower panel shows zoom from ROI. Scale bar, 50 µm. (F) Immunofluorescent staining for phospho-paxillin (green) and VE-cadherin (magenta) of HUVECs on an umbilical cord. ROI is indicated in the merge. Images are inverted for clarification. Lower panel shows zoom from ROI. Scale bar, 50 µm. [file Image_1.jpeg]

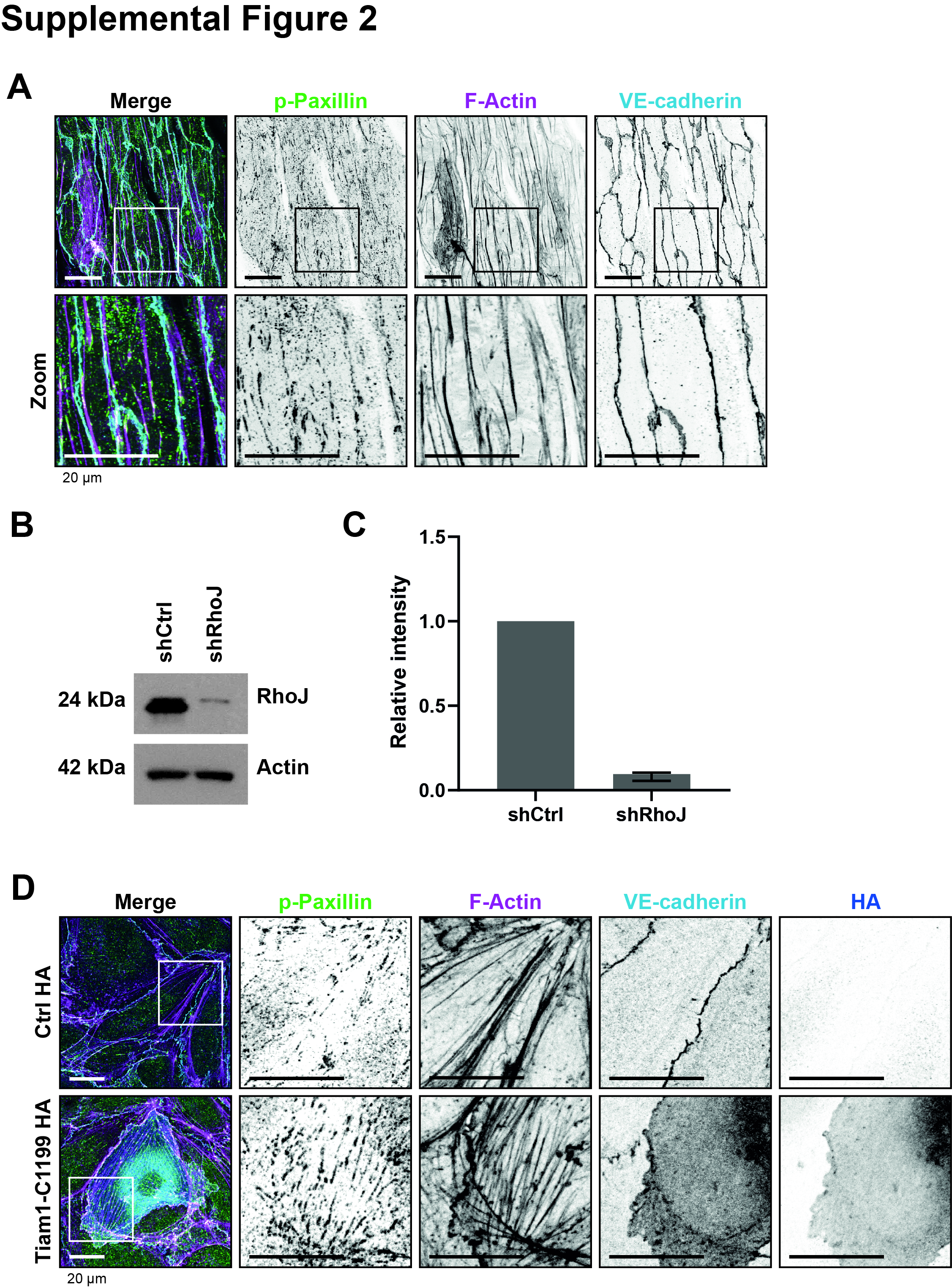

Supplement: Supplementary Figure 2 — (A) Immunofluorescent staining of mesenteric artery for phospho-paxillin (green), F-actin (magenta) and VE-cadherin (cyan). Lower panels are zoom of the ROI indicated in upper panels. Scale bar, 20 µm. (B) Western blot analysis shows successful knock down of RhoJ. Actin is shown as loading control. (C) Quantification of Western blot in (A). Error bar median with 95% confidence interval. Mann-Whitney U-test: p<0.05. (D) Immunofluorescent staining for phospho-paxillin (green), F-actin (magenta), VE-cadherin (Turquoise) and HA (blue) on HUVECs stimulated for 20h with TNFα. ROI is indicated in the merge. Images are inverted for clarification. Upper panel are control cells (Ctrl HA), lower panel are cells expressing Tiam1-C1199 (Tiam1-C1199). Scale bar, 20 µm. [file Image_2.jpeg]

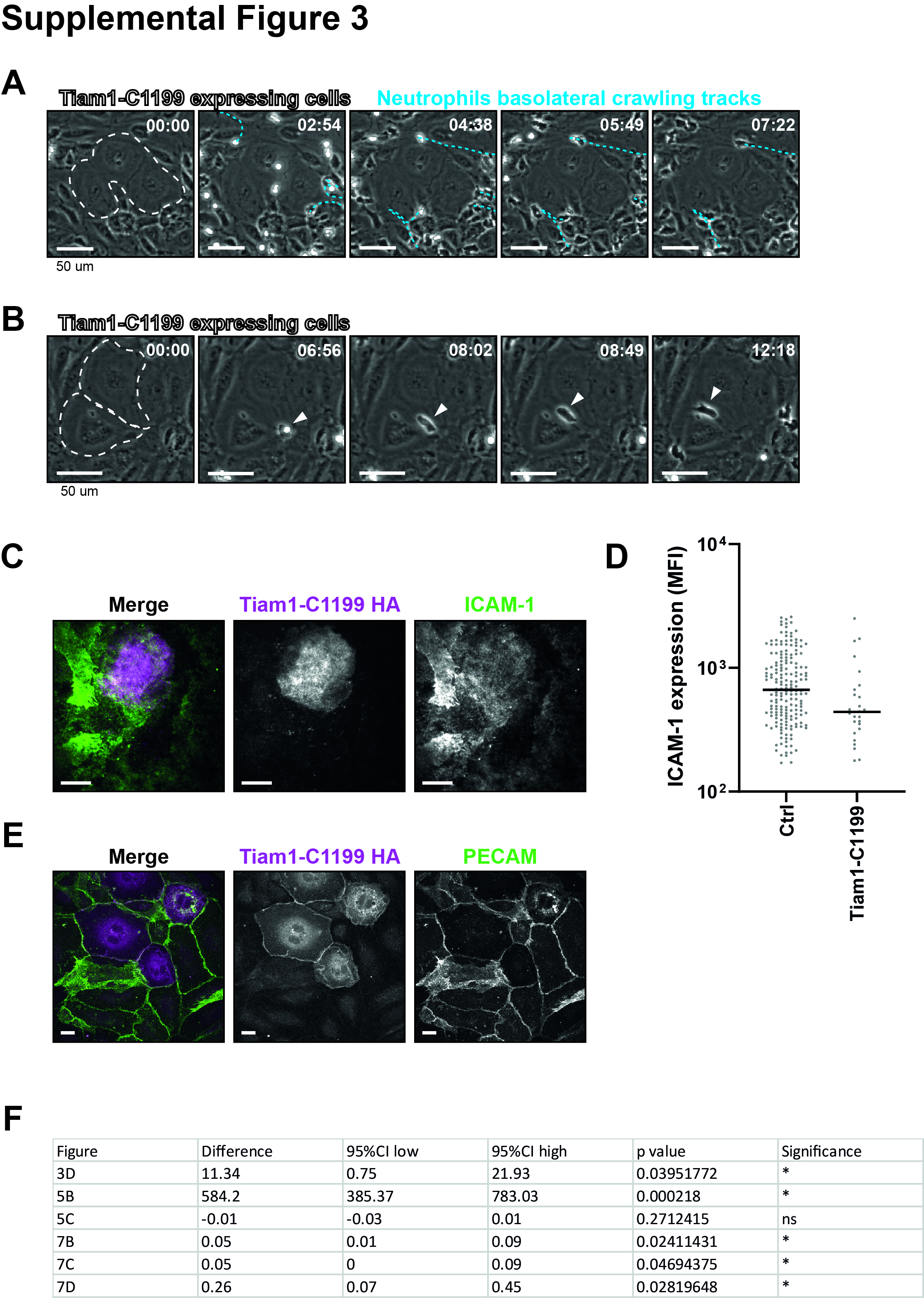

Supplement: Supplementary Figure 3 — (A) DIC stills from time lapse image showing three Tiam1-C1199 cells (dotted white line) and neutrophils that transmigrate at a ctrl-Tiam1-C1199 junction. Basolateral crawling tracks of these neutrophils are indicated in blue. Stills from Supplemental Movie 5. Time indicated in seconds in upper right corner. Bar, 50 μm. (B) DIC stills from time lapse image showing three Tiam1-C1199 cells (dotted white line) and a neutrophil that transmigrates at a ctrl-Tiam1-C1199 junction (arrowhead). The neutrophil remains stuck between the two Tiam1-C1199 cells. Stills from Supplemental Movie 6. Time indicated in seconds in upper right corner. Bar, 50 μm. (C) Immunofluorescent staining for ICAM-1 (green) and HA (Tiam1-C1199 HA) (magenta) on HUVECs stimulated for 4h with TNFα. ROI is indicated in the merge. Images are acquired using TIRF microscopy. Scale bar, 20 µm. (D) Quantification of ICAM-1 expression levels using MFI (mean fluorescence intensity) in HUVEC expressing Tiam1-C1199 or control (ctrl), all stimulated for 4h with TNFα. Lines indicate medians. (E) Immunofluorescent staining for PECAM (green) and HA (Tiam1-C1199 HA) (magenta) on HUVECs stimulated for 4h with TNFα. ROI is indicated in the merge. Images are acquired using confocal microscopy, maximum intensity projection. Scale bar, 20 µm. (F) Table with statistical information of the superplots ( Figures 3D , 5B , 5C , 7B , 7C , 7D ). [file Image_3.jpeg]
